# The influence of parental burnout on middle school students’ academic achievement: moderated mediation effect

**DOI:** 10.3389/fpsyg.2025.1530289

**Published:** 2025-02-03

**Authors:** Lingyi Peng, Huohong Chen, Jinhai Peng, Wei Liang, Mengfang Li, Weifeng Fu

**Affiliations:** ^1^Hunan First Normal University, Hunan, Changsha, China; ^2^Cognition and Human Behavior Key Laboratory, Hunan Normal University, Changsha, Hunan, China; ^3^Academic Affairs Office, Hunan Electronic Science and Technology Vocational College, Changsha, Hunan, China; ^4^Ma Tian Town Central Primary School, Chenzhou, China; ^5^Zhangjiang Town Middle School, Taoyuan, China

**Keywords:** parental burnout, academic achievement, academic self-efficacy, gender, middle school students, parental gender

## Abstract

**Introduction:**

This study investigates the influence of parental burnout on the academic achievement of middle school students, as well as the mediating role of academic self-efficacy and the moderating role of middle school students’ gender and parental gender.

**Methods:**

Utilizing a parent-child matched-pair design, a questionnaire survey was conducted with 738 middle school students and their parents (either fathers or mothers).

**Results:**

The findings revealed that: (1) parental burnout significantly and negatively predicted middle school students’ academic achievement; (2) academic self-efficacy partially mediated the relationship between parental burnout and middle school students’ academic achievement; and (3) the gender of middle school students moderated the initial segment of this mediating effect, while parental gender did not significantly moderate the relationship, indicating that the significant negative predictive effect of parental burnout on academic self-efficacy was evident only among female middle students.

**Discussion:**

These results not only enhance our understanding of the mechanisms and conditions under which parental burnout impacts middle school students’ academic achievement, but also have important implications for improving middle school students’ academic self-efficacy and overall academic performance.

## 1 Introduction

Since the 18th National Congress of the Communist Party of China, the country’s birth policy has evolved from “one couple, one child with exceptions” to “universal two-child” and now to “universal three-child.” While the relaxation of birth control policies has created more opportunities for reproduction, an increasing number of families remain hesitant to have additional children. Parenting issues have become a greater concern than the decision to expand family size (Zhong and Guo, 2017). It is widely acknowledged that the presence or arrival of children can bring immense joy and fulfillment to parents, but it also entails increased labor and sacrifice (Xu et al., 2018). Particularly when there is an imbalance between parenting demands and available resources, the phenomenon of parental burnout becomes highly prevalent (Mikolajczak et al., 2020). By definition, parental burnout shares similarities with occupational burnout and general burnout in that all are syndromes resulting from exposure to prolonged stress. However, it differs in that parental burnout arises specifically from the unique context of child-rearing (Isabelle et al., 2017). Parental burnout refers to a set of negative symptoms experienced by parents due to prolonged child-rearing stress. These symptoms include feelings of exhaustion in their parental role, a sense of discrepancy when comparing their current self to their former self as a parent, boredom with the parental role, and emotional distancing from their children (Roskam et al., 2018). Surveys indicate that the incidence of parental burnout in China ranges from 10 to 14% (Wang et al., 2021b). The negative consequences of general burnout are not only directed at the parents themselves but may also spill over to others. Due to the interactive nature of parenting, children are often the greater victims of general burnout (Cheng et al., 2021). Previous research has found that parental burnout is negatively correlated with children’s academic achievement (Hong et al., 2022). However, the underlying mechanisms of this relationship remain unclear. Therefore, this study aims to explore the internal mechanisms through which parental burnout affects children’s academic performance.

### 1.1 Parental burnout and academic achievement

Ecological systems theory posits that the family functions as a microsystem that directly and profoundly influences individual development (Bronfenbrenner, 1979). A positive family environment fosters individual development, whereas a detrimental environment has the opposite effect. Previous studies indicate that supportive parenting fosters emotional intelligence and adaptability in children, while also enhancing academic achievement by creating a learning environment characterized by open communication, encouragement, and shared decision-making. This, in turn, helps break the cycle of intergenerational poverty and promotes sustainable development (Ali et al., 2023; Tripon, 2024). Parental burnout, a family environmental factor that reflects negative emotions or attitudes related to parenting (Wang and Su, 2021), has been negatively correlated with children’s academic achievement (Hong et al., 2022). Parental burnout can result in a lack of parental fulfillment and self-doubt regarding parenting abilities (Mikolajczak et al., 2018), making it difficult to create an ideal family atmosphere. However, the family environment plays a more significant role than teacher-student relationships or classroom atmosphere in influencing adolescents’ academic achievement (Lei et al., 2012). Conversely, parents experiencing burnout are more likely to neglect or reject their children’s physiological and emotional needs (Roskam et al., 2017), and even increase the frequency of violent behaviors toward their children (Mikolajczak et al., 2018). Such negative and punitive parenting behaviors increase the risk of academic burnout in children (Luo et al., 2016), ultimately leading to a decline in academic performance. Moreover, parents severely affected by burnout find it difficult to provide not only basic parenting services but also additional resources for child-rearing, which may decrease their involvement in their children’s education. Negative parental involvement in education can also reduce children’s motivation to learn, thereby hindering their academic achievement (Yin et al., 2022). However, it is important to note that previous studies have primarily focused on elementary school students, while the rapid changes in the physical and psychological development of middle school students place higher demands on parental behavior (Wang et al., 2021b). Therefore, this study examines the impact of parental burnout on the academic achievement of middle school students.

### 1.2 The role of academic self-efficacy

Social cognitive theory posits that both environmental factors and individual characteristics (such as beliefs about one’s ability to complete specific tasks) jointly influence behavioral outcomes (Bandura, 1997). According to this theory, parental burnout, as an environmental factor, may affect middle school students’ academic achievement by influencing their academic self-efficacy. Academic self-efficacy refers to an individual’s judgment and confidence in their ability to complete academic tasks (Wang et al., 2016). As a specific manifestation of self-efficacy in the academic domain (Xiao and Liu, 2017), children’s academic self-efficacy is closely related to parental upbringing styles (Tang et al., 2014). Parenting style is a comprehensive concept that encompasses parents’ attitudes, beliefs, and behaviors in child-rearing (Zhang and Chen, 2013). Parenting styles are generally categorized into positive parenting (e.g., emotional warmth) and negative parenting (e.g., neglect or rejection), based on their impact on children’s positive development (Liu et al., 2021). Positive parenting creates a warm, supportive family environment and offers encouraging verbal persuasion, fostering higher levels of academic self-efficacy in children (Anna et al., 2017). Conversely, negative parenting often results in increased negative emotional arousal and experiences of failure, hindering the development of academic self-efficacy in children (Zhang and Ke, 2021). Research indicates that parents experiencing burnout often lose the patience necessary for effective child-rearing, attempt to evade their parental responsibilities, and neglect or reject their children’s requests for care (Cheng et al., 2021). Such neglect and rejection lower children’s self-confidence and impair their recognition of their own abilities (Fan and Williams, 2010). Moreover, parental burnout may affect children’s academic self-efficacy. Additionally, academic self-efficacy is a sensitive predictor of students’ academic performance (Lei et al., 2015). Yi et al. (2017) conducted a qualitative review and directional analysis of previous literature using meta-integration techniques, finding that among six factors (motivation, academic self-efficacy, achievement goal orientation, parenting style, teacher-student relationships, peer relationships), academic self-efficacy showed the highest correlation with students’ academic performance. Therefore, based on the above, academic self-efficacy may serve as a bridge between parental burnout and middle school students’ academic achievement.

### 1.3 The role of middle school students’ gender and parental gender

Resource dilution theory posits that as parenting stress increases, parents may become more cautious in allocating limited time, energy, and other resources (Blake, 1981). Consequently, parents who experience burnout due to excessive exposure to parenting stress may be able to provide only limited support and resources for their children. Additionally, patriarchal cultural norms contribute to a phenomenon known as “son preference” in China (Wu et al., 2013). Consequently, when resources are scarce, parents experiencing burnout may be more likely to favor their sons over their daughters, sacrificing the well-being of their daughters (Hannum et al., 2009). This results in less educational support, lower educational expectations, and reduced educational involvement for daughters (Liu et al., 2022), which is closely related to their academic self-efficacy (Zhou et al., 2023). On the other hand, girls exhibit higher empathy than boys (Zhang et al., 2020), making them more likely to detect their parents’ burnout early (e.g., low efficacy in parenting activities, disappointment in the parental role, emotional distance from children) and thus more affected by this imbalanced state. Research shows that individuals who grow up in environments characterized by parental neglect or indifference are less likely to recognize their own abilities (Zhang, 2022). Thus, the impact of parental burnout on middle school students’ academic self-efficacy may vary depending on the gender of the students. Furthermore, studies have shown that, compared to fathers, mothers are the primary caregivers in child-rearing activities (Cheng et al., 2021). Mothers tend to invest more energy in parenting and experience higher levels of parenting burnout (Norberg, 2010). As burnout increases, parents are more likely to exhibit negative parenting behaviors, such as neglect, rejection, or violence (Mikolajczak et al., 2018; Mikolajczak et al., 2019), which, in turn, affects their children’s academic self-efficacy (Tang et al., 2014). Therefore, the effect of parental burnout on middle school students’ academic self-efficacy may also differ based on the parent’s gender.

### 1.4 The present study

**Figure 1:**
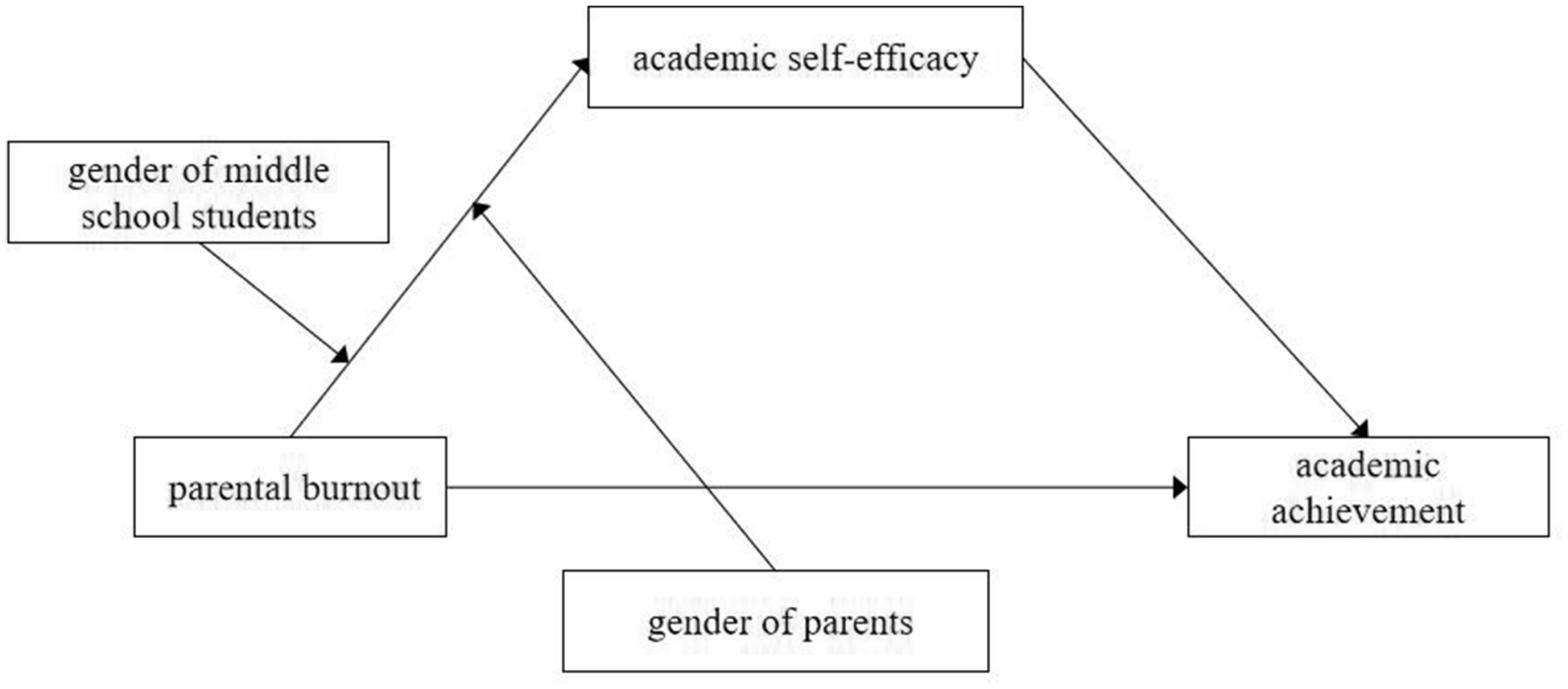
Hypothesized conceptual model.

Building on previous research and relevant theories, the present study aims to construct a moderated mediation model (see Figure 1) to examine the impact of parental burnout on middle school students’ academic achievement, as well as the mediating role of academic self-efficacy and the moderating effects of middle school students’ gender and parental gender. The specific hypotheses of this study are as follows:

*H1*: Parental burnout negatively predicts middle school students’ academic achievement.

*H2*: Academic self-efficacy mediates the relationship between parental burnout and middle school students’ academic achievement.

*H3*: Both the gender of middle school students and that of their parents moderate the effect of parental burnout on middle school students’ academic self-efficacy.

## 2 Methods

### 2.1 Participants

Participants in this study included students and their parents (i.e., either the father or mother) from a secondary school in Hunan, China. This investigation received the approval from the authors’ University Ethics Committee. Prior to data collection, the examiners (i.e., the head teacher of class who had undergone rigorous training in advance) introduced the instructions to participants and explained the principle of confidentiality. Participants were also informed that completing the survey was optional and that they could withdraw the investigation at any time. Once informed consent was obtained, participants were invited to fill out the questionnaires. Parents completed questionnaires during the parent-teacher meeting, while students completed theirs during the self-study class on the same day.

Based on Guo and Huang (2022), the sample size was calculated using the following formula $N=\frac{Z^{2}\times \left[P\times \left(1-P\right)\right]}{E^{2}}$ (N = sample size, Z = statistical value, E = margin of error, P = probability value). In this study, Z = 1.96 (95% confidence level), *E* = 5%, *P* = 12.20% (based on previous research indicating that the incidence of parental burnout in China ranges from 10.44 to 13.95%; Wang et al., 2021b). The minimum required sample size for this study was therefore determined to be 165. The collected data were first matched based on the names, classes, and genders of both parents’ reports on their children and the students’ self-reports. Only the matched data were retained. Subsequently, questionnaires with missing answers or those exhibiting patterned responses were further excluded. Ultimately, we obtained valid paired data for 738 parent–child dyads, indicating a sufficient sample size for the study. Among the parent data, 184 were fathers (24.9%) and 554 were mothers (75.1%), with an average age of 43.21 ± 4.37 years. The sample included 310 single-child families (42.0%), 389 two-child families (52.7%), 35 three-child families (4.7%), and 4 four-child families (0.5%). Among the children, there were 239 males (32.4%) and 499 females (67.6%), with 309 attending junior high school (41.9%) and 429 attending senior high school (58.1%), and an average age of 14.87 ± 1.47 years.

### 2.2 Measures

#### 2.2.1 Parental burnout scale

The parental burnout scale developed by Roskam et al. (2018) was utilized. This scale consists of 23 items across four dimensions: exhaustion in the parental role (e.g., “I really do not know how to raise my children anymore”), comparison with past parental roles (e.g., “To play the role of a good parent, I am exhausted”), boredom with the parental role (e.g., “I do not want to take on the role of a parent again”), and emotional distancing from the child (e.g., “I can no longer be a good parent”). Items are rated on a 7-point scale ranging from “*never*” to “*every day*,” with higher scores indicating higher levels of parental burnout. The scale has been widely used in different cultural contexts (e.g., Mikolajczak et al., 2019; Roskam et al., 2021; Sorkkila and Aunola, 2020), and the Chinese version has been validated with good reliability (Yang et al., 2021). In this study, the Cronbach’s *α* coefficient for the parental burnout scale was 0.91.

#### 2.2.2 Academic self-efficacy scale

The academic self-efficacy Scale developed by Liang (2000) was used. This scale includes 22 items across two dimensions: academic ability self-efficacy (e.g., “I believe I have the ability to achieve good grades in my studies”) and academic behavioral self-efficacy (e.g., “I often cannot accurately summarize the main points of what I read”). Items are rated on a 5-point scale ranging from “strongly disagree” to “strongly agree,” with higher scores indicating stronger academic self-efficacy. The scale has demonstrated good reliability and validity in previous research (Ye et al., 2017). In this study, the Cronbach’s α coefficient for the Academic Self-Efficacy Scale was 0.90.

#### 2.2.3 Academic achievement

To assess students’ academic achievement, we used subject grades as the evaluation indicator. Since Chinese, Math, and English are considered core subjects in China’s education system, previous studies have primarily assessed students’ academic achievement based on the grades of these core subjects (Lei et al., 2015; Qiao et al., 2013). Following this practice, the participants’ scores in Chinese, Math, and English from the monthly exams conducted during data collection were obtained through communication with the class teachers. These scores were standardized within the grade level. The overall academic achievement index was calculated as the average of the standardized scores in the three subjects (Lei et al., 2015).

#### 2.2.4 Demographic variables

We also collected relevant demographic information from both parents and children. Specifically, parents were asked to report their gender, age, number of children, as well as their children’s names, class, and gender. Students were asked to report their name, class, gender, and age. These demographic variables were collected at the beginning of the formal testing.

#### 2.2.5 Data analyses

Descriptive statistics, correlation analysis, common method bias testing, and model testing for mediation and moderation effects were performed using SPSS 26.0 and PROCESS v3.5 (Hayes, 2013).

## 3 Results

### 3.1 Common method bias

Given that this study utilized a questionnaire method, there was a potential risk of common method bias. To mitigate the influence of this bias on the research outcomes, procedural controls were implemented, including the use of highly reliable and valid measurement tools and emphasizing confidentiality. Statistically, Harman’s single-factor test was employed to assess common method bias. The results indicated that there were 8 factors with eigenvalues greater than 1, and the variance explained by the first factor was 25.51%, which is below the critical value of 40%. This suggests that the issue of common method bias in this study is not severe (Podsakoff et al., 2003).

### 3.2 Descriptive statistics and Pearson correlation analysis

Table 1 presents the means, standard deviations, and Pearson correlation coefficients for the variables. The Pearson correlation analysis revealed that parental burnout was significantly negatively correlated with academic self-efficacy (*r* = −0.19, *p* < 0.001) and academic achievement (*r* = −0.15, *p* < 0.001). Academic self-efficacy was positively correlated with academic achievement (*r* = 0.26, *p* < 0.001). Additionally, the gender of middle school students was positively correlated with parental burnout (*r* = 0.09, *p* < 0.05) and academic self-efficacy (*r* = 0.12, *p* < 0.01), but negatively correlated with academic achievement (*r* = −0.08, *p* < 0.05). However, neither the gender of parents nor the number of children exhibited significant correlations with these variables (*r* = −0.04 ~ 0.05, *p* > 0.05).

**Table 1:** Descriptive statistics of variables and Pearson correlation matrix (*N* = 738).

Variable	*M* ± *SD*	1	2	3	4	5
1. Parental burnout	1.58 ± 0.65					
2. Academic self-efficacy	3.14 ± 0.58	−0.19^***^				
3. Academic achievement	0.01 ± 0.79	−0.15^***^	0.26^***^			
4. Gender of middle school students	**—**	0.09^*^	0.12^**^	−0.08^*^		
5. Gender of parents	**—**	0.01	0.01	−0.01	0.08^*^	
6. Number of children	**—**	0.05	−0.01	0.03	−0.08^*^	−0.04

The gender of the middle school students, gender of parents, and number of children are all dummy variables: 1 = male, 0 = female; 1 = only child, 0 = two or more children; 1 = father, 0 = mother; ^*^p < 0.05, ^**^p < 0.01, ^***^p < 0.001, and so on.

### 3.3 Moderated mediation analysis

Testing a moderated mediation model involves two steps: first, examining a simple mediation model, and second, examining a moderated mediation model (Wen and Ye, 2014). For this study, bias-corrected non-parametric percentile Bootstrap was used for testing, with 5,000 resamples to compute the 95% confidence interval. Before conducting the mediation and moderated mediation analyses, all variables were standardized.

#### 3.3.1 Step 1

Examining the mediating role of academic self-efficacy (Model 4). The results showed that parental burnout significantly negatively predicted both academic achievement (*c* = −0.12, *p* < 0.001) and academic self-efficacy (*a* = −0.19, *p* < 0.001). When both parental burnout and academic self-efficacy were entered into the regression equation, parental burnout continued to significantly negatively predicted academic achievement (*c’* = −0.09, *p* < 0.01), while academic self-efficacy significantly positively predicted academic achievement (*b* = 0.19, *p* < 0.001). The indirect effect (ab) was −0.04, with *Boot SE* = 0.01, and the 95% confidence interval was [−0.07, −0.01], which does not include zero. This indicates that academic self-efficacy partially mediates the relationship between parental burnout and academic achievement. The proportion of the indirect effect (*ab*) relative to the total effect (*c*) was −0.04/−0.12 = 33.33%.

#### 3.3.2 Step 2

**Figure 2:**
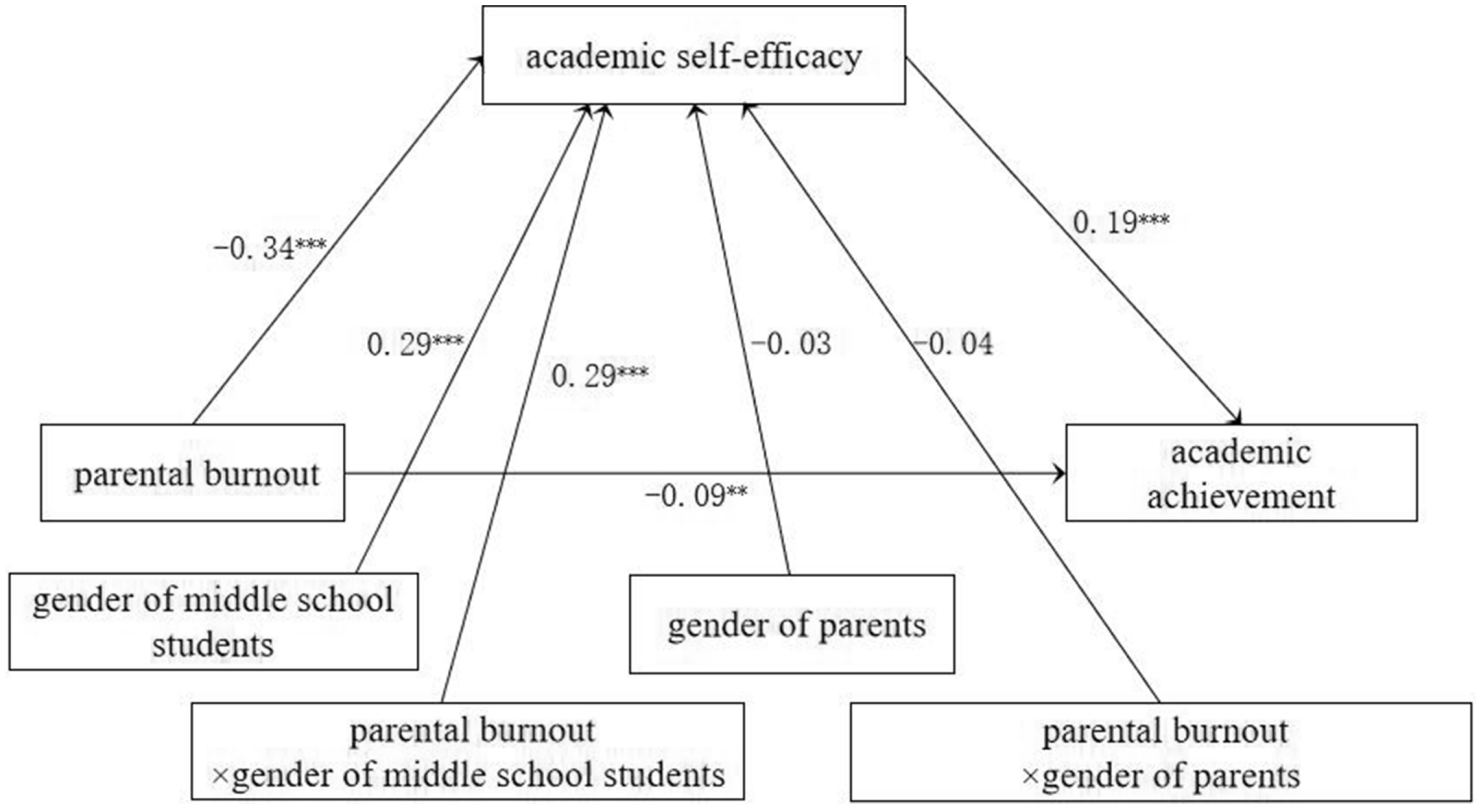
Moderated mediation effect test (*N* = 738).

Including the gender of middle school students and parents in the model to analyze the moderated mediation effect (model 9). The results (see Figure 2) showed that parental burnout (*β* = −0.34, *p* < 0.001) and the gender of middle school students (*β* = 0.29, *p* < 0.001) significantly predicted academic self-efficacy, and the interaction term between parental burnout and the gender of middle school students also significantly predicted academic self-efficacy (*β* = 0.29, *p* < 0.001). However, the gender of parents (*β* = −0.03, *p* > 0.05) did not significantly predict academic self-efficacy, nor did the interaction between parental burnout and the gender of parents did not significantly predict academic self-efficacy (*β* = −0.04, *p* > 0.05). Both academic self-efficacy (*β* = 0.19, *p* < 0.001) and parental burnout (*β* = −0.09, *p* < 0.01) significantly predicted academic achievement. This suggests that gender of middle school student moderates the first part of the mediation pathway (parental burnout → academic self-efficacy → academic achievement), whereas the gender of parents does not moderate this pathway.

**Figure 3:**
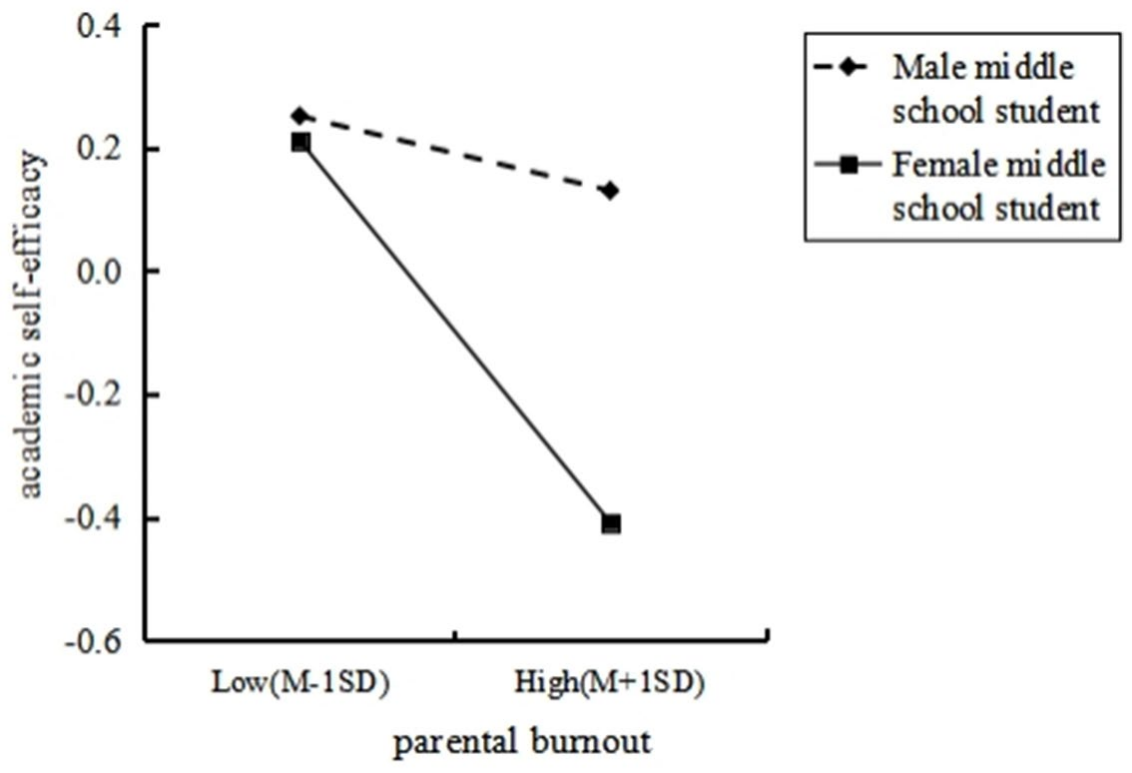
The moderating role of middle school students’ gender on the relationship between parental burnout and academic self-efficacy.

To further test the moderating effect, the interaction plot for the gender of middle school students (male middle school student = 1, female middle school student = 0) was created (see Figure 3). The slope of the lines in the plot reflects the influence of parental burnout on academic self-efficacy. Simple slopes analysis showed that for female middle school students, as parental burnout increased, their academic self-efficacy significantly decreased (*b*_simple_ = −0.34, *t* = −6.52, *p* < 0.001). For male middle school students, however, parental burnout did not significantly predict their academic self-efficacy (*b*_simple_ = −0.07, *t* = −1.29, *p* > 0.05).

## 4 Discussion

This study elucidates the relationship between parental burnout and middle school students’ academic achievement, as well as the mechanisms through which this relationship operates. Specifically, it explains how parental burnout affects academic performance—namely, through the mediating role of academic self-efficacy—and when it affects performance, highlighting gender differences in the mediating effect that is evident only among female middle school students.

### 4.1 The influence of parental burnout on middle school students’ academic achievement

The study found that parental burnout significantly negatively predicts middle school students’ academic achievement, consistent with findings from studies involving elementary school students (Hong et al., 2022). Parents experiencing burnout often lack emotional engagement with their children and may resort to punitive parenting behaviors (Mikolajczak et al., 2018). When middle school students face academic challenges, burned-out parents may either ignore their requests for help or resort to punitive discipline. Both of these negative parenting practices can undermine students’ motivation for academic progress and adversely affect their academic performance (Wang et al., 2020; Yin et al., 2022). Therefore, parents should recognize that an imbalanced parenting state is closely linked to negative academic outcomes for middle school students. While parental stress is entirely normal, it is crucial for parents to manage and alleviate this stress, keeping it within manageable limits to create a supportive environment for their children’s development (Yu, 2021).

### 4.2 The mediating role of academic self-efficacy

Furthermore, the study found that academic self-efficacy plays a partial mediating role in the relationship between parental burnout and middle school students’ academic performance. This result supports the social cognitive theory (Bandura, 1997). As the level of parental burnout increases, parents’ interest and motivation in their children’s education decline (Yu, 2021), leading to reduced parental involvement. When parents cease to assist with homework or fail to communicate their educational expectations, this directly lowers children’s academic self-efficacy (Cross et al., 2019; Williams et al., 2017). Additionally, academic self-efficacy, as a specific manifestation of general self-efficacy in the academic domain (Xiao and Liu, 2017), indicates that students with low academic self-efficacy tend to view academic tasks, difficulties, and setbacks as obstacles and are less likely to adopt problem-focused coping strategies when faced with academic pressure (Denovan and Macaskill, 2017). Consequently, they are less likely to perform well on exams (Lei et al., 2015). Thus, maintaining a balanced parenting state can provide stronger support for children’s academic success. Positive educational interactions can help children recognize their learning abilities, leading to better academic outcomes.

### 4.3 The moderating role of middle school students’ gender and parental gender

The study also found that the gender of middle school students moderates the first half of the mediating effect of academic self-efficacy in the relationship between parental burnout and academic performance. Specifically, in female middle school students, increased parental burnout leads to a decrease in academic self-efficacy, which in turn hinders their academic performance; this indirect effect is not observed in male middle school students. Parents experiencing burnout often reduce the parenting resources available to their children (Roskam et al., 2017), particularly the educational support that children expect. Moreover, compared to boys, parents may assign lower educational expectations and involvement to girls due to the perceived lower returns on educational investment for girls in the labor market (Liu et al., 2022). Girls who perceive a reduction in parental educational involvement may experience a decrease in their recognition of their own learning behaviors and capabilities (Liu et al., 2018), which can negatively influence their academic performance. It is also important to note that the insignificant negative predictive effect of parental burnout on academic self-efficacy in male middle school students does not necessarily imply that this effect is absent in this group. There may be cumulative effects, and future research should employ longitudinal designs to further validate these findings. Additionally, the study found that parental burnout’s effect on students’ academic self-efficacy was not moderated by parental gender. This could be related to changes in family division of labor. As societal norms evolve, the traditional “male breadwinner, female homemaker” model is gradually diminishing, with more women entering the workforce and more men participating in parenting activities (Gao et al., 2020; Wang et al., 2021a). This shared parenting responsibility may have balanced parental burnout between fathers and mothers (Delvecchio et al., 2015), rendering the effect of parental burnout on students’ academic self-efficacy independent of parental gender.

### 4.4 Implications and limitations

This study constructed a moderated mediation model to explore the relationship between parental burnout and the academic achievement of secondary school students, along with the mediating role of academic self-efficacy and the moderating roles of student and parental gender. The findings provide theoretical guidance and empirical support for enhancing secondary school students’ academic achievement. On one hand, intervening in parental burnout is a crucial starting point. Some researchers suggest that strengthening mutual support and cooperation among family members can effectively alleviate parental burnout (Yu, 2021). This includes shared family parenting responsibilities (Lin et al., 2023), joint parenting by grandparents and parents (Yu, 2021), and fostering children’s self-management abilities to reduce dependence on their parents (Li et al., 2024). Additionally, schools and communities could offer training to improve parents’ time management skills, guide them in setting realistic parenting goals, developing scientific parenting plans, prioritizing tasks, and reflecting on their parenting behaviors (Huang and Zhang, 2001), which could enhance the quality of family parenting and reduce parental burnout. Moreover, establishing a family-school-community collaborative education mechanism, optimizing national parental welfare policies, and encouraging companies to develop family-friendly policies (Ann and Harrington, 2007) can also reduce workplace stress and parental burnout. On the other hand, for students experiencing parental burnout, schools should focus on enhancing their academic self-efficacy, particularly among female students. Strategies such as providing opportunities for students to experience success, promoting role models, offering timely positive feedback, focusing on study skills, creating a supportive learning environment, implementing rational attribution training, and encouraging extracurricular physical activities (Bandura, 1997; Mcauley et al., 2000; Olivier et al., 2019; Zhu, 2012) can all boost students’ academic confidence.

At the same time, this study has some limitations. First, the cross-sectional design limits the ability to make causal inferences. Future studies could explore this relationship through longitudinal tracking designs. Second, the data used in this study were all self-reported by the participants, which may have introduced response biases. For the variable of parental burnout, future research could benefit from incorporating both parent and child reports to mitigate response bias and improve the stability of the measurements (Song, 2023). Third, the participants selected for this study were only middle school students and their parents from a specific region, without involving other areas. Future studies should examine middle school students and their parents from a broader geographic range. Forth, this study only considered the number of children as a control variable, while factors such as family socioeconomic status and family structure are also important influences on parental burnout (Baxter et al., 2014). Finally, this study only explored a limited set of mediating and moderating variables in the relationship between parental burnout and students’ academic achievement. Future research could further examine other potential mediators and moderators, such as parental involvement, teacher support, and students’ emotions and motivation.

## 5 Conclusion

Academic self-efficacy partially mediates the relationship between parental burnout and middle school students’ academic achievement. Furthermore, this mediating effect is moderated by the gender of the middle school students but not by the gender of the parents. Specifically, the negative influence of parental burnout on academic self-efficacy is significant only among female middle school students.

## Data Availability

The raw data supporting the conclusions of this article will be made available by the authors, without undue reservation.
